# A fast direct solver for surface-based whole-head modeling of transcranial magnetic stimulation

**DOI:** 10.1038/s41598-023-45602-5

**Published:** 2023-10-31

**Authors:** S. N. Makaroff, Z. Qi, M. Rachh, W. A. Wartman, K. Weise, G. M. Noetscher, M. Daneshzand, Zhi-De Deng, L. Greengard, A. R. Nummenmaa

**Affiliations:** 1 Electrical and Computer Engineering Department, Worcester Polytechnic Institute, Worcester, MA 01609 USA; 2 Athinoula A. Martinos Ctr. for Biomedical Imaging, Massachusetts General Hospital, Harvard Medical School, Charlestown, MA 02129 USA; 3 Center for Computational Mathematics, Flatiron Institute, New York, NY 10010 USA; 4 Max Planck Institute for Human Cognitive and Brain Sciences, Stephanstr. 1a, 04103 Leipzig, Germany; 5 Advanced Electromagnetics Group, Technische Universität Ilmenau, Helmholtzplatz 2, 98693 Ilmenau, Germany; 6 Computational Neurostimulation Research Program, Noninvasive Neuromodulation Unit, Experimental Therapeutics and Pathophysiology Branch, National Institute of Mental Health, NIH 10 Center Drive, Bethesda, MD 20892 USA; 7 Courant Institute of Mathematical Sciences, 251 Mercer Street, New York, NY 10012 USA

**Keywords:** Computational neuroscience, Biophysical models, Software

## Abstract

When modeling transcranial magnetic stimulation (TMS) in the brain, a fast and accurate electric field solver can support interactive neuronavigation tasks as well as comprehensive biophysical modeling. We formulate, test, and disseminate a direct (i.e., non-iterative) TMS solver that can accurately determine global TMS fields for any coil type everywhere in a high-resolution MRI-based surface model with ~ 200,000 or more arbitrarily selected observation points within approximately 5 s, with the solution time itself of 3 s. The solver is based on the boundary element fast multipole method (BEM-FMM), which incorporates the latest mathematical advancement in the theory of fast multipole methods—an FMM-based LU decomposition. This decomposition is specific to the head model and needs to be computed only once per subject. Moreover, the solver offers unlimited spatial numerical resolution. Despite the fast execution times, the present direct solution is numerically accurate for the default model resolution. In contrast, the widely used brain modeling software SimNIBS employs a first-order finite element method that necessitates additional mesh refinement, resulting in increased computational cost. However, excellent agreement between the two methods is observed for various practical test cases following mesh refinement, including a biophysical modeling task. The method can be readily applied to a wide range of TMS analyses involving multiple coil positions and orientations, including image-guided neuronavigation. It can even accommodate continuous variations in coil geometry, such as flexible H-type TMS coils. The FMM-LU direct solver is freely available to academic users.

## Introduction

Transcranial magnetic stimulation (TMS) is a non-invasive technique used to stimulate specific regions of the brain. For TMS to be a more effective and personalized therapeutic tool, it is crucial to accurately simulate the induced electric field (E-field) distribution in the brain. For interactive E-field based TMS neuronavigation, the E-field should be updated and displayed fast enough to provide the operator with a sense of real-time interaction.

There are several time scales to consider. First, most real-time visualization applications have a minimum update rate of around 15–30 frames per second (fps) to have a smooth and continuous experience; this allows for approximately 30–60 ms for coil position update, E-field solution update, and image rendering. The second time scale of relevance relates to the pace at which a human or a robot^1, 2^ could move a TMS coil from one position to another. Given an estimate for sequential robotic targeting speed of ~ 1 cm/s^1^, and if we consider coil position change of a few millimeters to produce a meaningful change in the E-field, then the solution can be updated on the time scale on the order of 100 ms. The final time scale is that of the TMS interstimulus interval, if the E-field solution were to update synchronous with TMS pulse delivery. For conventional motor mapping with single pulses, an approximately ~ 5 s interstimulus interval is typical to avoid inhibitory effects (cf^3, 4^).

Over the past two years, significant progress has been made with regard to excellent real-time high-resolution solvers for TMS field modeling^5–7^, which execute in approximately 20–50 ms. These simulation speeds are close to the standard screen refresh rate and should be very useful in practice for continuous smooth field visualization. In general, these solvers do not involve accelerating the main numerical algorithm itself but rather apply highly efficient and “smart” interpolation methods to thousands of precomputed solution sets.

This study aims to introduce a direct accurate TMS solver that can output TMS fields from any coil and at any location in a high-resolution head model within ~ 3–5 s. This solver is based on the boundary element fast multipole method or BEM-FMM^8–11^ and the most recent mathematical advance in the theory of fast multipole methods—an FMM-based LU factorization or FMM-LU^12^. It does not use any interpolation routines or precomputed solutions, but does require initial LU decomposition of a head-model matrix, which takes approximately 40 min.

One motivation for this study is a modern precise motor mapping protocol^13^, which would benefit from fast accurate field calculations while the participant is still in the hospital or facility so that a follow up TMS session could be avoided. In addition, the mapping process could be further optimized through adaptive algorithms that will utilize a sequential approach for estimating the “hot spot”^14^. On the other hand, the fast solver can be readily utilized to pre-compute a large number of E-field solutions covering “all possible coil positions/orientations” of interest that can be subsequently used to stimulate a desired cortical target derived from anatomical or functional connectivity data (see, e.g.,^15^). The precalculated solutions could also be used to guide the operator to the right target in an interactive neuronavigation setting.

In contrast to the finite element method, BEM formulations are restricted to the surface of compartment domains, thereby reducing the dimensionality of the problem for brain modeling in general. There has been a resurgence of interest in these surface-based methods due to the availability of fast algorithms such as fast multipole methods (FMMs)^16, 17^. Assuming *N* is the number of degrees of freedom used in sampling surfaces and $A$ is the $N\times N$ system matrix ($Ax=b$) obtained after the application of a suitable quadrature rule to an integral representation, these algorithms permit $A$ to be applied to a vector in $O(N)$ or $O(N logN)$ time. For well-conditioned systems, this allows for the rapid iterative solution of very large-scale problems. The recently developed charge-based boundary element fast multipole method (BEM-FMM)^8–11^ utilizes this type of iterative solution. It requires 20–50 iterations on average and executes in 30–60 s depending on the required accuracy. These data are for high-resolution surface-based head models such as those generated by FreeSurfer^18, 19^ and SPM/CAT^20^.

There is an obvious task where iterative solvers are not satisfactory. This includes near real-time E-field predictions for TMS brain mapping or stimulation planning with a large number of possible coil positions, orientations, or even coil geometry changes. Each such setup gives rise to a unique right-hand side. Here, direct solvers would be preferred to solve the same system matrix with *multiple* right-hand sides. Along with this, fast direct solvers could be useful when exploring low-rank perturbations of the head geometry (and/or tissue conductivities) and, hence, the system matrix. Updating the solution in such cases requires only a few applications of ${A}^{-1}$ or a fast update of the inverse itself^21, 22^.

In the last few years, several algorithmic ideas have emerged which permit the construction of a compressed approximation of ${A}^{-1}$ at a cost of the order $O(N)$ or $O(N{log}_{p}N)$, for modest *p*. To construct the first fast direct TMS solver, we apply one such scheme^12^, which is referred to as the FMM-LU method^12^. It uses FMM-type hierarchical compression strategies to rapidly compute an LU-factorization of the large system matrix.

## Materials and methods

### Charge-based BEM in a succinct form

Induced electric charges with a surface charge density $\rho ({\varvec{r}})$ will reside on every tissue conductivity interface $S$ once an external electromagnetic stimulus or a primary E-field ${{\varvec{E}}}^{p}\left({\varvec{r}}\right)$ of a TMS coil is applied. These induced surface charges will alter the primary field to fulfill the law of current conservation across the boundaries. The E-field generated by all surface charges anywhere in space except the charged interfaces themselves is governed by Coulomb’s law. The total E-field ${\varvec{E}}\left({\varvec{r}}\right)$ becomes the sum of the primary field and the secondary charge field i.e.,

1 $${\varvec{E}}\left( {\varvec{r}} \right) = {\varvec{E}}^{p} \left( {\varvec{r}} \right) + \frac{1}{{4\pi \varepsilon_{0} }}\mathop \int \limits_{S}^{{}} \frac{{{\varvec{r}} - \user2{r^{\prime}}}}{{\left| {{\varvec{r}} - \user2{r^{\prime}}} \right|^{3} }}\rho \left( {\user2{r^{\prime}}} \right)d\user2{r^{\prime}},\quad {\varvec{r}} \notin S$$

where ${\varepsilon }_{0}$ is dielectric permittivity of vacuum (a normalization constant). The E-field in Eq. (1) is discontinuous at the interfaces. When approaching a charged interface $S$ with a certain normal vector ${\varvec{n}}$ and assigning index *into* the medium from which ${\varvec{n}}$ is pointing and index *out* of the medium toward which ${\varvec{n}}$ is pointing, the E-field close to the boundary is given by two limiting values^23^

2 $${\varvec{E}}_{in/out} \left( {\varvec{r}} \right) = {\varvec{E}}^{p} \left( {\varvec{r}} \right) + \frac{1}{{4\pi \varepsilon_{0} }}\mathop \int \limits_{S}^{{}} \frac{{{\varvec{r}} - \user2{r^{\prime}}}}{{\left| {{\varvec{r}} - \user2{r^{\prime}}} \right|^{3} }}\rho \left( {\user2{r^{\prime}}} \right)d\user2{r^{\prime}} \mp {\varvec{n}}\left( {\varvec{r}} \right)\frac{{\rho \left( {\varvec{r}} \right)}}{{2\varepsilon_{0} }},\quad {\varvec{r}} \in S$$

where ${\varvec{E}}_{in/out} \left( {\varvec{r}} \right) \equiv \mathop {\lim }\nolimits_{\Delta \to 0} {\varvec{E}}\left( {{\varvec{r}} \mp \Delta {\varvec{n}}\left( {\varvec{r}} \right)} \right)$ and ${\varvec{r}} \mp \Delta {\varvec{n}}\left( {\varvec{r}} \right)$ is not on the surface. The second term on the right-hand side of Eq. (2) is a continuous contribution of all other surface charges while the last term is a discontinuous contribution of a local planar sheet of charge located exactly at ${\varvec{r}}$ resulting in a jump of the normal E-field by $\rho \left({\varvec{r}}\right)/{\varepsilon }_{0}$. The “discrete” interpretation of this jump relation of the potential theory^23, 24^ is that the integral on the right-hand side of Eq. (2) is the continuous contribution of surface charges of all facets except the facet located exactly at ${\varvec{r}}$ while the last term on its right-hand side is a discontinuous contribution of the facet located exactly at ${\varvec{r}}$. This facet is a planar sheet of charge.

An integral equation for $\rho ({\varvec{r}})$ is obtained after substitution of Eq. (2) into the quasistatic boundary condition which enforces the continuity of the normal component of the electric current across the interface. That is,

3 $$\sigma_{in} {\varvec{n}}\left( {\varvec{r}} \right) \cdot {\varvec{E}}_{in} \left( {\varvec{r}} \right) = \sigma_{out} {\varvec{n}}\left( {\varvec{r}} \right) \cdot {\varvec{E}}_{out} \left( {\varvec{r}} \right),\quad {\varvec{r}} \in S$$

here ${\sigma }_{in},{\sigma }_{out}$ are the conductivities just inside and outside with respect to the direction of the normal vector. After combining similar terms, a Fredholm equation of the second kind is obtained

4 $$\frac{{\rho \left( {\varvec{r}} \right)}}{2} - K\left( {\varvec{r}} \right){\varvec{n}}\left( {\varvec{r}} \right) \cdot \mathop \int \limits_{S}^{{}} \frac{1}{4\pi }\frac{{{\varvec{r}} - \user2{r^{\prime}}}}{{\left| {{\varvec{r}} - \user2{r^{\prime}}} \right|^{3} }}\rho \left( {\user2{r^{\prime}}} \right)d\user2{r^{\prime}} = K\left( {\varvec{r}} \right){\varvec{n}}\left( {\varvec{r}} \right) \cdot {\varvec{E}}^{p} \left( {\varvec{r}} \right), \quad {\varvec{r}} \in S$$

where $K$ is the electric conductivity contrast $K=\frac{{\sigma }_{in}-{\sigma }_{out}}{{\sigma }_{in}+{\sigma }_{out}}$ for the facet positioned at ${\varvec{r}}$.

We emphasize that the present formulation is a multi-compartment BEM. The number of head compartments with different conductivity (and conductivity contrast) values is not limited. For example, BEM-FMM routinely handles head models with 7–16 compartments^8–11, 25^. It can also handle a head model with 116 compartments^26^, and can include surrounding space of an unlimited extent^11^. However, BEM computations for high-resolution multi-compartment head models are only possible with a fast multipole method (FMM) acceleration^8–11, 25^. The BEM-FMM formulation (largely the involved FMM formulation) is more difficult to implement numerically than the standard FEM approach. However, easy-to-use FMM libraries are available^27^.

### Iterative (BEM-FMM) versus direct (FMM LU) solution of Eq. (4)

Assuming that the charge density $\rho ({\varvec{r}})$ has a constant value ${x}_{m}$ for every triangular facet ${t}_{m}$ with area ${A}_{m}$, Eq. (4) is discretized in standard matrix form (${{\varvec{E}}}_{{\varvec{m}}}^{p}$ is the primary field at the *m*-th facet)

5 $$Ax = b, \quad A_{mn} = \frac{1}{2}\delta_{mn} - \frac{K}{{A_{m} }}{\varvec{n}}_{m} \cdot \iint\limits_{{t_{m} t_{n} }} {\frac{1}{4\pi }\frac{{\left( {{\varvec{r}} - \user2{r^{\prime}}} \right)}}{{\left| {{\varvec{r}} - \user2{r^{\prime}}} \right|^{3} }}d\user2{r^{\prime}}d{\varvec{r}}},\user2{ }\quad b_{m} = \frac{K}{{A_{m} }}{\varvec{n}}_{m} \cdot {\varvec{E}}_{{\varvec{m}}}^{p}$$

where ${\delta }_{mn}$ is Kronecker delta. Matrix $A$ is well conditioned. Therefore, an iterative solution of Eq. (5) with the generalized minimum residual method (GMRES)^28^ converges fast, in 20–40 iterations. FMM is used to compute the matrix–vector product in a “matrix free” fashion so that matrix $A$ is never computed/stored^27, 29^. This solution was used previously^8–11^.

Another way to solve Eq. (5) is to apply FMM to a direct LU-decomposition of matrix $A$. In other words, a compressed approximation of ${A}^{-1}$ is found directly so that a direct solution $x={A}^{-1}b$ is attempted, without using an iterative method and for multiple right-hand sides, $b$. While matrix $A$ is determined by the head model, the right-hand sides describe coil fields. This is the compression algorithm of Ref.^12^, which is applied in this study. Indeed, the compressed inverse differs from the iterative solution. We will show that this difference is vanishingly small.

### Human models and coil models used to test the algorithm

First, we tested the default example of FEM software SimNIBS v3.2.6^30^—the Ernie head model (~ 850,000 facets) with the default Magstim 70 mm coil model targeting the ${M1}_{\mathrm{HAND}}$ area of the left hemisphere and thoroughly described in^31^ (“Supplementary Information”). The MRI segmentation was done with the headreco pipeline^31^, which uses the SPM12/CAT^32^ toolbox. The region of interest (ROI) is a part of the midsurface between white and gray matter within a sphere with a radius of 2 cm centered at the target.

Second, we tested four Connectome Young Adult^33^ subjects and again targeted the ${M1}_{\mathrm{HAND}}$ area of the left hemisphere with the MRiB91 coil of MagVenture using sulcus-aligned coil positioning^34^. All head models have been obtained via.

(i) *FreeSurfer* segmentation^18, 19^ (seven compartments, ~ 830,000 facets each) and;
(ii) *Headreco* segmentation^31^ (seven compartments, ~ 1,050,000 facets each).

**Figure 1:**
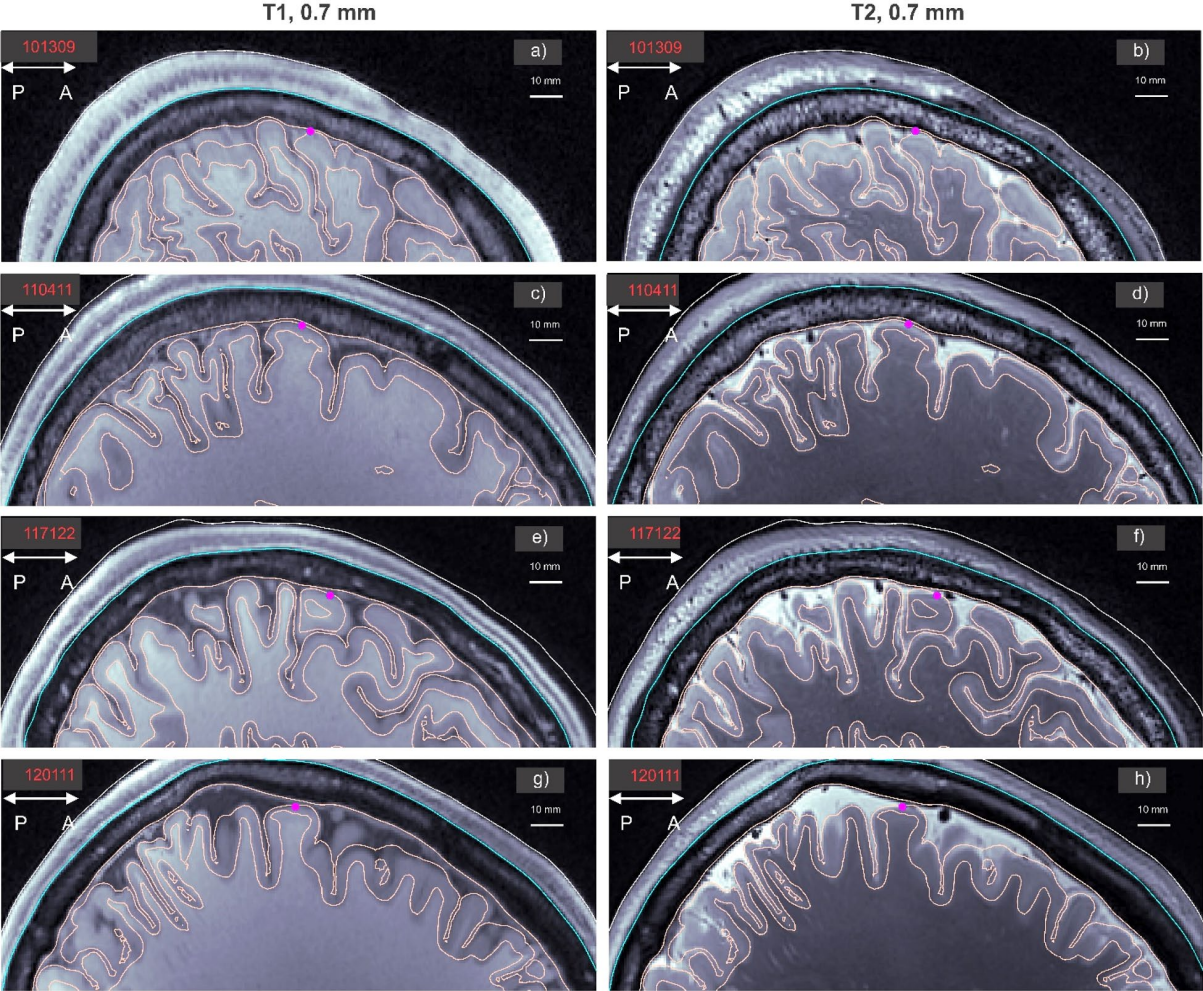
T1 and T2 MRI data with 0.7 mm isotropic resolution for 4 Human Connectome subjects overlapped with the *headreco* segmentation used in this study in a sagittal plane passing through the target point at the approximate center of the ${\mathrm{M}1}_{\mathrm{HAND}}$ area of the left hemisphere. The ${\mathrm{M}1}_{\mathrm{HAND}}$ target is a circle. Each segmentation model used for computations includes seven 2-manifold compartments: skin or scalp (yellow), skull or bone (cyan), cerebrospinal fluid or CSF (pale pink), gray matter or GM (pale pink), white matter or WM (pale pink), ventricles (not seen), and eyes (not seen). All compartments have been kept.

Figure 1 shows T1 and T2 MRI data, both with 0.7 mm isotropic resolution, for four Human Connectome subjects overlapped with the *headreco* segmentation used in this study in a sagittal plane passing through the target point at the approximate center of the ${\mathrm{M}1}_{\mathrm{HAND}}$ area of the left hemisphere. The ${\mathrm{M}1}_{\mathrm{HAND}}$ target in Fig. 1 is a small magenta circle.

Each *headreco* segmentation model from Fig. 1 used for computations includes seven 2-manifold compartments: skin or scalp (yellow), skull or bone (cyan), cerebrospinal fluid or CSF (pale pink), gray matter or GM (pale pink), white matter or WM (pale pink), ventricles (not seen), and eyes (not seen). All compartments were retained when performing computations.

Similarly, each *FreeSurfer* segmentation model used for computations includes seven slightly different 2-manifold compartments: skin or scalp, skull, CSF, GM, WM, ventricles, and cerebellum. All compartments were retained when performing computations as well.

The TMS coil was approximated by ~ 30,000-elementary current elements (with the skin effect included) and the coil fields were also computed via the fast multipole method^35^. The ROI is a part of the midsurface between white and gray matter within a sphere with the radius of 2 cm centered at the target.

**Figure 2:**
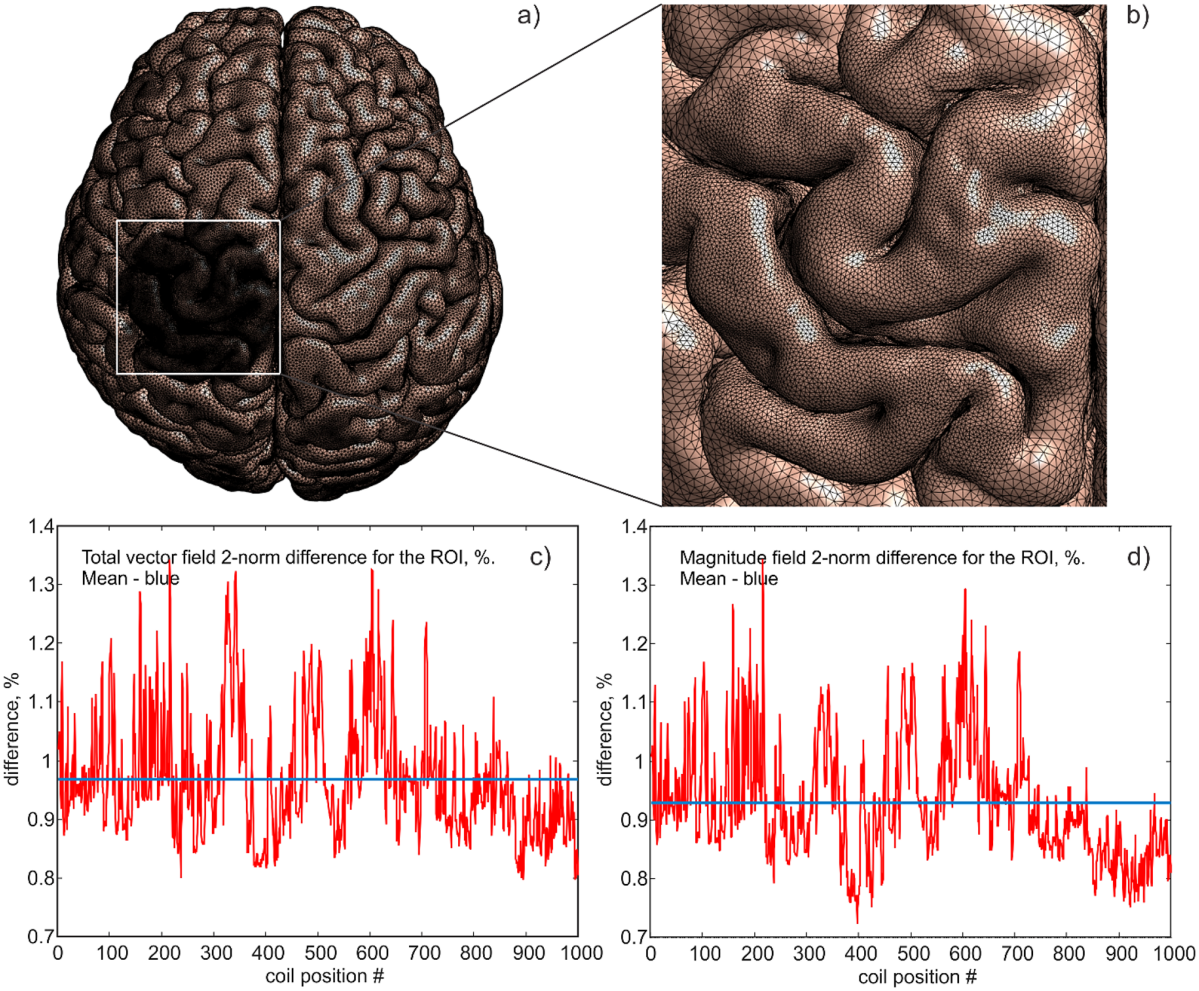
*Headreco* segmentation of the MPI Leipzig healthy subject in (**a**) was manually refined for cerebrospinal fluid, gray matter, and white matter within the ROI in (**a**,**b**). One thousand different coil positions have been tested while scanning the ROI; SimNIBS and BEM-FMM solutions were compared with each other in (**c**,**d**).

Finally, we tested a healthy subject scanned at Max Planck Inst. for Human Cogn. & Brain Sciences Leipzig, Germany with the MagVenture CB65 coil and with the same left ${M1}_{\mathrm{HAND}}$ target. However, the corresponding *headreco* model with 7 compartments was manually refined in the region of interest (ROI) as illustrated in Fig. 2 so that the overall model size is now ~ 1,800,000 facets. In all cases above, the default SimNIBS conductivity parameters^31^ have been used.

### Testing method

Both accuracy and speed were tested. Two error types were considered: the relative error in the vector E-field, ${Error}_{total}$, and the relative error in the magnitude of the E-field, ${Error}_{mag}$

6 $$Error_{total} = \frac{{\left\| {{\varvec{E}}_{t} \left( {\varvec{r}} \right) - \user2{E^{\prime}}_{t} \left( {\varvec{r}} \right)} \right\|}}{{\left\| {{\varvec{E}}_{t} \left( {\varvec{r}} \right)} \right\|}},\quad Error_{mag} = \frac{{\left\| {\left| {{\varvec{E}}_{t} \left( {\varvec{r}} \right)} \right|{ } - \left| {\user2{E^{\prime}}_{t} \left( {\varvec{r}} \right)} \right|} \right\|}}{{\left\| {\left| {{\varvec{E}}_{t} \left( {\varvec{r}} \right)} \right|} \right\|}}$$

over an arbitrary domain of interest where $\left\| \cdot \right\|$ is a 2-norm for a vector or scalar field. Index $t$ denotes the total E-field in the head which is a sum of the primary field and the secondary field.

## Reference solution

As a reference (or “ground truth”) solution, an iterative high-resolution BEM-FMM solution has been chosen, also following the approach of Ref.^36^. We mention several reasons justifying this choice:

(i) When comparing with an analytical solution for a four-layer sphere, BEM–FEM is 10 times or even more accurate than the first-order FEM (of SimNIBS)^25, 36^ when the same computational mesh is used. This observation is to be expected since FEM must additionally mesh an otherwise piecewise homogeneous volume and thus introduce extra numerical error.
(ii) Both FEM and BEM-FMM converge to the same result when the FEM volumetric (and surface) mesh is additionally refined. In Fig. 2a,b. the default SimNIBS *headreco* segmentation^31^ of the healthy subject was manually refined for cerebrospinal fluid, gray matter, and white matter (both surface and volume meshes were refined) as shown in Fig. 2b. One thousand different coil positions (using the MagVenture CB65 coil) were tested while scanning the ROI. A SimNIBS solution using the refined mesh and the BEM-FMM solution were compared with each other as shown in Fig. 2c,d, respectively. The average vector field and magnitude field differences from Eq. (6) within the ROI in Fig. 2c,d now attain *sub-percent* values, which confirms the convergence of both methods to the same result.
(iii) For current piecewise homogeneous head models, it is possible to achieve the BEM-FMM numerical resolution of 0.1–0.2 mm or better while the corresponding FEM meshes would be prohibitively large in size.

Thus, an iterative BEM-FMM solution with 54 million facets (a 1:64 uniform mesh refinement of all seven head compartments for the Ernie model, average edge length of 0.18 mm), with the FMM internal precision^27^ of 1e−6, and with a terminal residual of 1e-6 has been utilized as the reference solution in the following Table 1.

**Table 1:** Field error values from Eq. (6) for the mid-surface between gray and white matter.

Error types and their percentage values	SimNIBS Ernie model (%)	1:4 Uniform Refinement	1:16 Uniform Refinement
${Error}_{total}$ for entire midsurface/SimNIBS FEM	27.1	–	–
${Error}_{total}$ for entire midsurface/iterative BEM-FMM	14.7	1.7%	0.5%
${Error}_{total}$ for entire midsurface/BEM-FMM LU	14.7	–	–
${Error}_{total}$ for 4 cm ROI under coil/SimNIBS FEM	25.0	–	–
${Error}_{total}$ for 4 cm ROI under coil/iterative BEM-FMM	3.3	0.4%	0.1%
${Error}_{total}$ for 4 cm ROI under coil/BEM-FMM LU	3.3	–	–
${Error}_{mag}$ for entire midsurface /SimNIBS FEM	17.7	–	–
${Error}_{mag}$ for entire midsurface/iterative BEM-FMM	4.9	0.6%	0.2%
${Error}_{mag}$ for entire midsurface/BEM-FMM LU	4.9	–	–
${Error}_{mag}$ for 4 cm ROI under coil/SimNIBS FEM	5.7		
${Error}_{mag}$ for 4 cm ROI under coil/iterative BEM-FMM	1.6	0.2%	0.06%
${Error}_{mag}$ for 4 cm ROI under coil/BEM-FMM LU	1.6	–	–

The results for midsurface nodes are reported, which are nearly undistinguishable (difference of 0.1% or less) from the results for centers of the midsurface facets. The default Ernie model of the SimNIBS v3.2.6 FEM software with the default headreco segmentation and the default coil type/position is tested. Three solutions are considered: the default SimNIBS FEM solution, the iterative BEM-FMM solution, and the new BEM-FMM LU solution. When an outlier removal with 0.1% of the largest local errors being removed was applied, the total field error from the second row of Table 1 reduced from 27.1 to 26.2% and the ROI field error from the fifth row of Table 1 reduced from 25.0 to 22.8%

## Results

### Method accuracy versus SimNIBS FEM accuracy

For the default Ernie model example^31^ of the FEM SimNIBS software^30^, Table 1 gives the field error values for a mid-surface between the gray and white matter with ~ 200,000 observation nodes. The results for midsurface nodes are reported here, which are nearly undistinguishable (difference of 0.1% or less) from the results for centers of the midsurface facets. Three solutions were considered: the default SimNIBS FEM solution, the iterative BEM-FMM solution, and the direct FMM LU solution—the subject of this study. For the iterative BEM-FMM solution, we also provide results for the uniformly refined models.

When an outlier removal with 0.1% of the largest local errors being removed was applied, the SimNIBS total field error from the second row of Table 1 reduced from 27.1 to 26.2% and the SimNIBS ROI field error from the fifth row of Table 1 reduced from 25.0 to 22.8%.

The iterative BEM-FMM solution in Table 1 uses the FMM precision of 1e−4 and the terminal residual of 1e−6. The FMM LU uses the same FMM precision of 1e−4. Two measures of the error are given: one is for the entire midsurface between gray and white matter while the second one covers a region of interest (ROI) on the midsurface contained within a 4 cm diameter sphere at the target point on the coil axis crossing the gray matter interface.

### Method accuracy at different levels of internal FMM precision

Table 2 gives the field error values for the mid-surface between gray and white matter. Two solutions were compared against each other: the iterative BEM-FMM solution and the BEM-FMM LU solution. The iterative BEM-FMM uses the FMM precision of 1e−4 everywhere, including the field computations, and the terminal residual of 1e−6. The FMM LU solution was tested with different values of FMM accuracy for (i) decomposition; and (ii) field computations. Four Connectome Young Adult models, subjects 101,309, 110,411, 117,122, 120,111^33^, were tested when the MRiB91 coil of MagVenture was targeting the ${M1}_{\mathrm{HAND}}$ of the left hemisphere. Both *FreeSurfer* and *headreco* segmentations with seven compartments each were studied. Along with this, the Ernie model provided with SimNIBS FEM software, segmented with the standard headreco settings, and the default coil type/position was also tested. The superscript in Table 2 indicates standard deviation.

**Table 2:** Field error values from Eq. (6) for the mid-surface between gray and white matter.

Error type and percentage values	SimNIBS Ernie model (%)	4 Connectome models, averaged *headreco* seg	4 Headreco models, averaged *FreeSurfer* seg
FMM LU precision: 1e−4: Field FMM precision: 1e−4			
${Error}_{total}$ for entire midsurface/BEM-FMM LU versus iterative sol	0.10	0.11%^0.02%^	0.12%^0.02%^
${Error}_{total}$ for 4 cm ROI under coil/BEM-FMM LU versus iterative sol	0.03	0.03%^0.03%^	0.05%^0.02%^
${Error}_{mag}$ for entire midsurface/BEM-FMM LU versus iterative sol	0.08	0.08%^0.01%^	0.09%^0.01%^
${Error}_{mag}$ for 4 cm ROI under coil/BEM-FMM LU versus iterative sol	0.03	0.02%^0.005%^	0.05%^0.02%^
FMM LU precision: 1e−4: Field FMM precision: 1e−1			
${Error}_{total}$ for entire midsurface/BEM-FMM LU versus iterative sol	0.37	0.38%^0.02%^	1.34%^0.29%^
${Error}_{total}$ for 4 cm ROI under coil/BEM-FMM LU versus iterative sol	0.15	0.16%^0.02%^	0.40%^0.08%^
${Error}_{mag}$ for entire midsurface/BEM-FMM LU versus iterative sol	0.28	0.27%^0.02%^	1.00%^0.15%^
${Error}_{mag}$ for 4 cm ROI under coil/BEM-FMM LU versus iterative sol	0.15	0.14%^0.01%^	0.28%^0.03%^
FMM LU precision: 1e−3: Field FMM precision: 1e−1			
${Error}_{total}$ for entire midsurface/BEM-FMM LU versus iterative sol	1.35	1.46%^0.29%^	0.90%^0.98%^
${Error}_{total}$ for 4 cm ROI under coil/BEM-FMM LU versus iterative sol	0.40	0.42%^0.16%^	0.20%^0.21%^
${Error}_{mag}$ for entire midsurface/BEM-FMM LU versus iterative sol	1.05	1.04%^0.19%^	0.61%^0.69%^
${Error}_{mag}$ for 4 cm ROI under coil/BEM-FMM LU versus iterative sol	0.39	0.37%^0.18%^	0.15%^0.16%^

The FMM-LU is tested with different values of FMM precision for (i) LU matrix decomposition and; (ii) field computations. Four Connectome models (with both FreeSurfer and Headreco segmentations, respectively) are tested along with the default Ernie model of the SimNIBS FEM software. The superscript denotes standard deviation.

### Method speed

**Figure 3:**
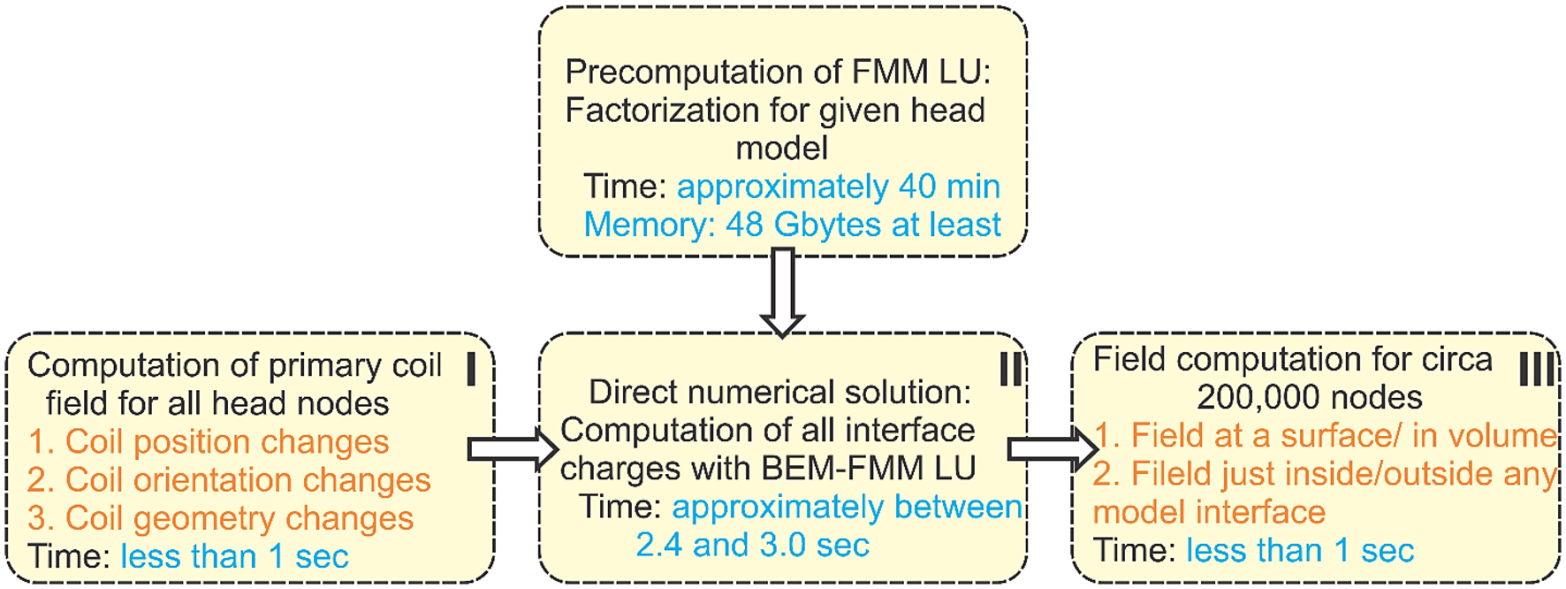
Approximate execution times of the method when the parameters from Table 2 are used for the FMM-LU solution (the Ernie model). The data are averaged for four workstations with Intel(R) Xeon(R) Gold 6348 CPU @ 2.60 GHz 512 GB RAM, 56 cores, OS Windows Server 2022 Standard; MATLAB 2021b platform. The entire solution executes in slightly less than 5 s.

The method speed was tested using four nearly identical workstations with Intel(R) Xeon(R) Gold 6348 CPU @ 2.60 GHz, 512 GB RAM, 56 cores, OS Windows Server 2022 Standard and the common MATLAB 2022b platform. Figure 3 outlines the corresponding execution times including precomputations for the Ernie model with the standard options. In Fig. 3, initial computations of the primary coil field and the final field computations for the given domain (steps I and III) take less than 1 s for the FMM precision levels 1e−1 and 1e−2 while the direct numerical solution (step II) takes 2.4 s for the FMM precision level 1e−3 and 3.0 s for the FMM precision level 1e−4, respectively.

Equivalent SimNIBS v3.2.6/4.0 execution times on high-performance workstations were approximately 40 s. The SimNIBS run times on a 2.3 GHz laptop are faster—approximately 30 s.

Further experimentations with *FreeSurfer* and *headreco* segmentations, respectively, for four Connectome subjects from Table 2 have shown that the method execution time and memory (RAM) consumption increase nearly linearly with the model size (the number of facets), in line with the theoretical predictions^12^.

### Example #1. Cortical and subcortical fields computed in 4.7 s (MRiB91 coil)

**Figure 4:**
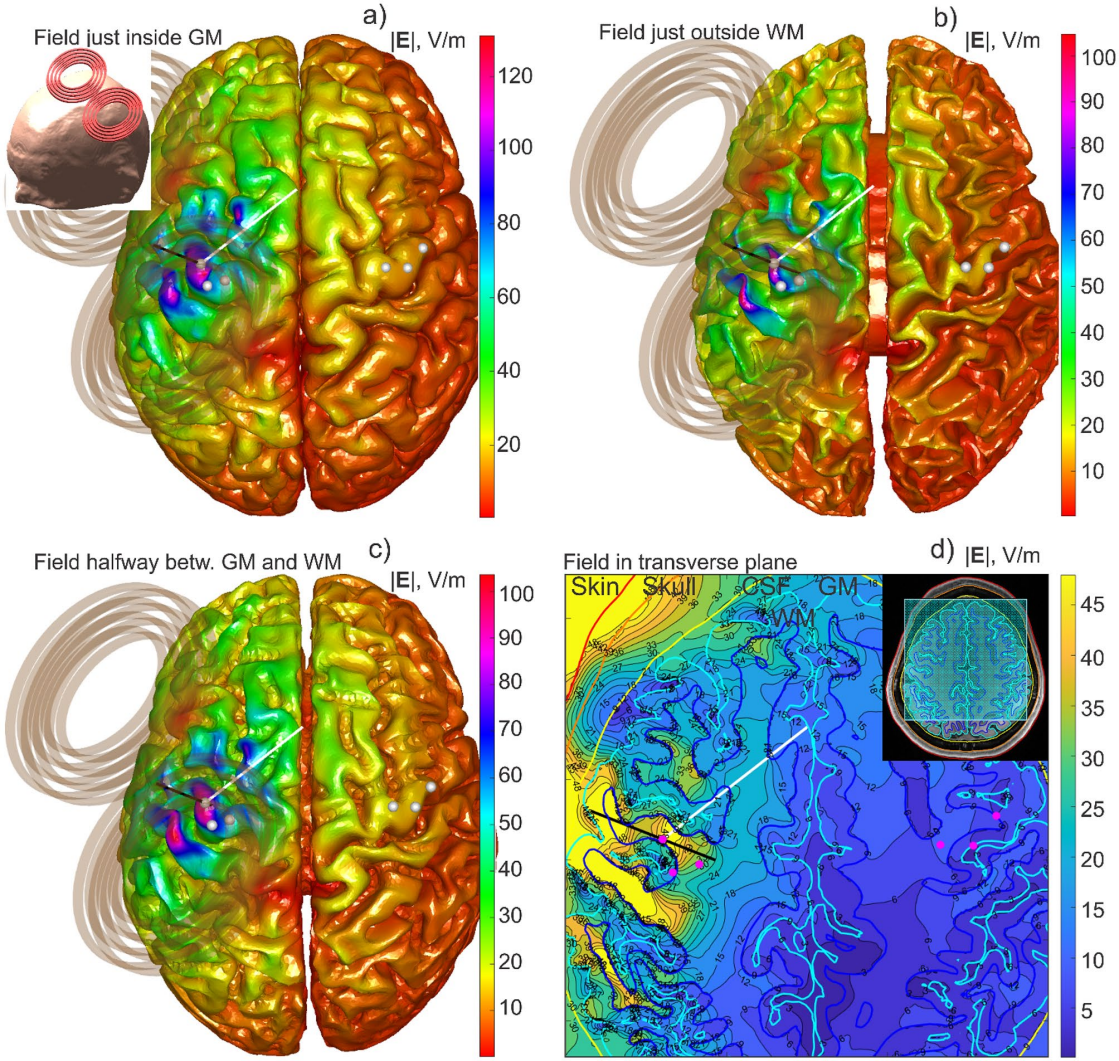
Different forms of the simulation output for Connectome subject 120,111. The MRiB91 coil of MagVenture was driven with $dI/dt=9.4e7 \mathrm{A}/\mathrm{s}$. (**a**) Total E-field just inside the gray matter interface; (**b**) Total E-field just outside the white matter interface; (**c**) Total E-field at the cortical midsurface; (**d**) Total E-field in a transverse plane beneath the coil. The entire computational sequence for any type of the output runs in approximately 4.7 s including graphical rendering in MATLAB.

As a first computational example, Fig. 4 shows the simulation output for Connectome Young Adult subject 120,111 including: (a) the total E-field just inside the gray matter interface; (b) the total E-field just outside the white matter interface; (c) the total E-field at the cortical midsurface and; (d) the total E-field in a transverse plane beneath the coil. All seven compartments of the *headreco* segmentation have been used, but the outermost compartments were not shown for clarity. The MRiB91 coil of MagVenture was driven with $dI/dt=9.4e7 \mathrm{A}/\mathrm{s}$.

The entire computational sequence for any type of output from Fig. 4 takes approximately 4.7 s to run (for the *FreeSurfer* segmentation), including graphical rendering in MATLAB. For the *headreco* segmentation, this number increases by ~ 25–30%.

### Example #2. Cortical and subcortical fields computed in 4.8 s (authentic H1 coil)

**Figure 5:**
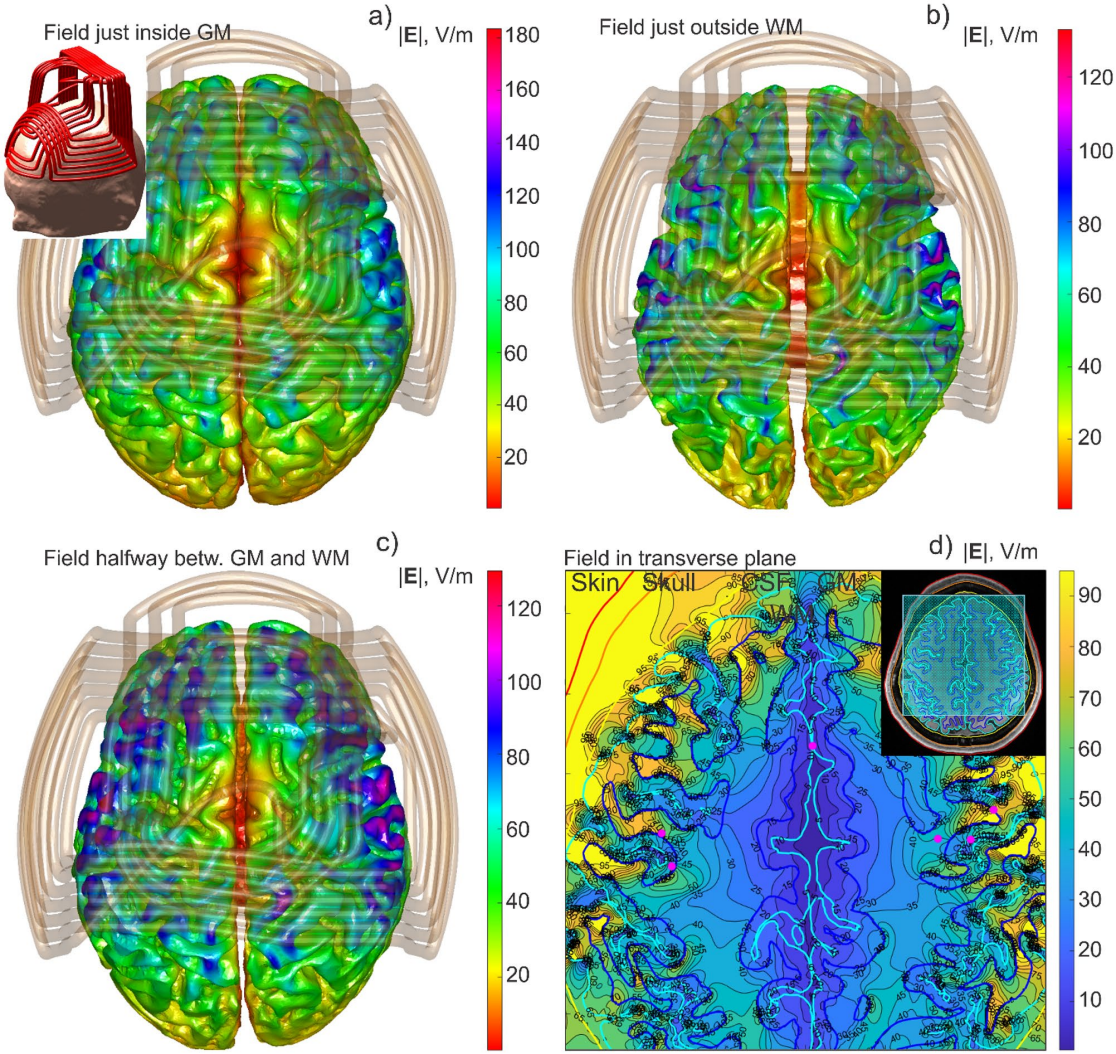
Different forms of the simulation output for Connectome subject 120,111 with the H1 flexible coil of BrainsWay, Ltd. The H1 coil was driven with $dI/dt=9.4e7 \mathrm{A}/\mathrm{s}$. (**a**) Total E-field just inside the gray matter interface; (**b**) Total E-field just outside the white matter interface; (**c**) Total E-field at the cortical midsurface; (**d**) Total E-field in a transverse plane beneath the coil. The entire computational sequence for any type of the output runs in approximately 4.8 s including graphical rendering in MATLAB.

As a second example, Fig. 5 shows a simulation output for the same Connectome subject 120,111 but when an H1 coil of BrainsWay is used. The H1 coil was again driven with $dI/dt=9.4e7 \mathrm{A}/\mathrm{s}$.

In this case, the coil is made *flexible*, and it changes its shape when continuously aligned with the patient’s head. Therefore, step I in Fig. 5 cannot be replaced by an interpolation of the precomputed coil field.

The entire computational sequence for any type of output from Fig. 5 takes approximately 4.8 s to run (for the *FreeSurfer* segmentation), including graphical rendering in MATLAB. For the *headreco* segmentation, this number increases by ~ 25–30%.

## Discussion

### Iterative and direct FMM solutions produce nearly identical results for various head segmentation models

Table 2 indicates that the accuracy of FMM LU is *non-distinguishable* from the accuracy of BEM-FMM given the FMM precision level of 1e−4 or better used for constructing the compressed inverse. In this case, the factorization data stored in MATLAB workspace have the size of approximately 50 Gigabytes; their creation requires approximately 70 min. The direct solution—step II in Fig. 3—executes in 3.0 s.

The FMM precision level used for the field computations—steps I and III in Fig. 3—does not affect data storage but influences speed. As Table 2 shows, it may be as low as 1e−1 without deteriorating the solution accuracy significantly.

When the FMM-LU precision level is reduced to 1e−3, the factorization data will have the size of 30 Gigabytes, their generation requires approximately 40 min, and the direct solution (step II in Fig. 3) executes in 2.4 s. This option could likely be preferred since its accuracy reported in the last four rows of Table 2 does not exceed 0.4% on average for the critical ROI fields.

As Table 2 demonstrates, the FMM precision level used for the field computations has little influence on the solution accuracy in the ROI itself. This is because in both BEM-FMM and FMM LU, the near-field interaction integrals are computed analytically and then substituted into the FMM pipelines. On the other hand, this level has a substantial influence on the method speed. Therefore, the values of 1e−2 or 1e−1 could likely be preferred.

### Solid agreement with SimNIBS is achieved when the SimNIBS FEM head model is refined

The lower 1st-order FEM accuracy observed in Table 1 can be improved using selective mesh refinement in the ROI domain as it was previously illustrated in Fig. 2a,b. Here, the default headreco segmentation^31^ of the healthy subject was manually refined for cerebrospinal fluid, gray matter, and white matter (both surface and volume mesh were refined) as shown in Fig. 2b. One thousand different coil positions (using a MagVenture CB65 coil) have been tested while scanning the ROI. Solutions using the refined meshes from SimNIBS and BEM-FMM were compared with each other as shown in Fig. 2c,d, respectively. 

The average vector field and magnitude field differences from Eq. (6) within the ROI in Fig. 2c,d attain the sub-percent values, which indicates that the refined FEM model does provide the adequate result. Due to the differences in the 2D versus 3D numerical formulations of the problems for the BEM-FMM and SIMNIBS, respectively, some FEM-caused discrepancies remain close to the conductivity boundaries where the normal electric field is discontinuous. The practical implications of these discrepancies require further investigation.

### Solid agreement with SimNIBS is achieved for activating thresholds of intracortical neural cells for the refined head model

**Figure 6:**
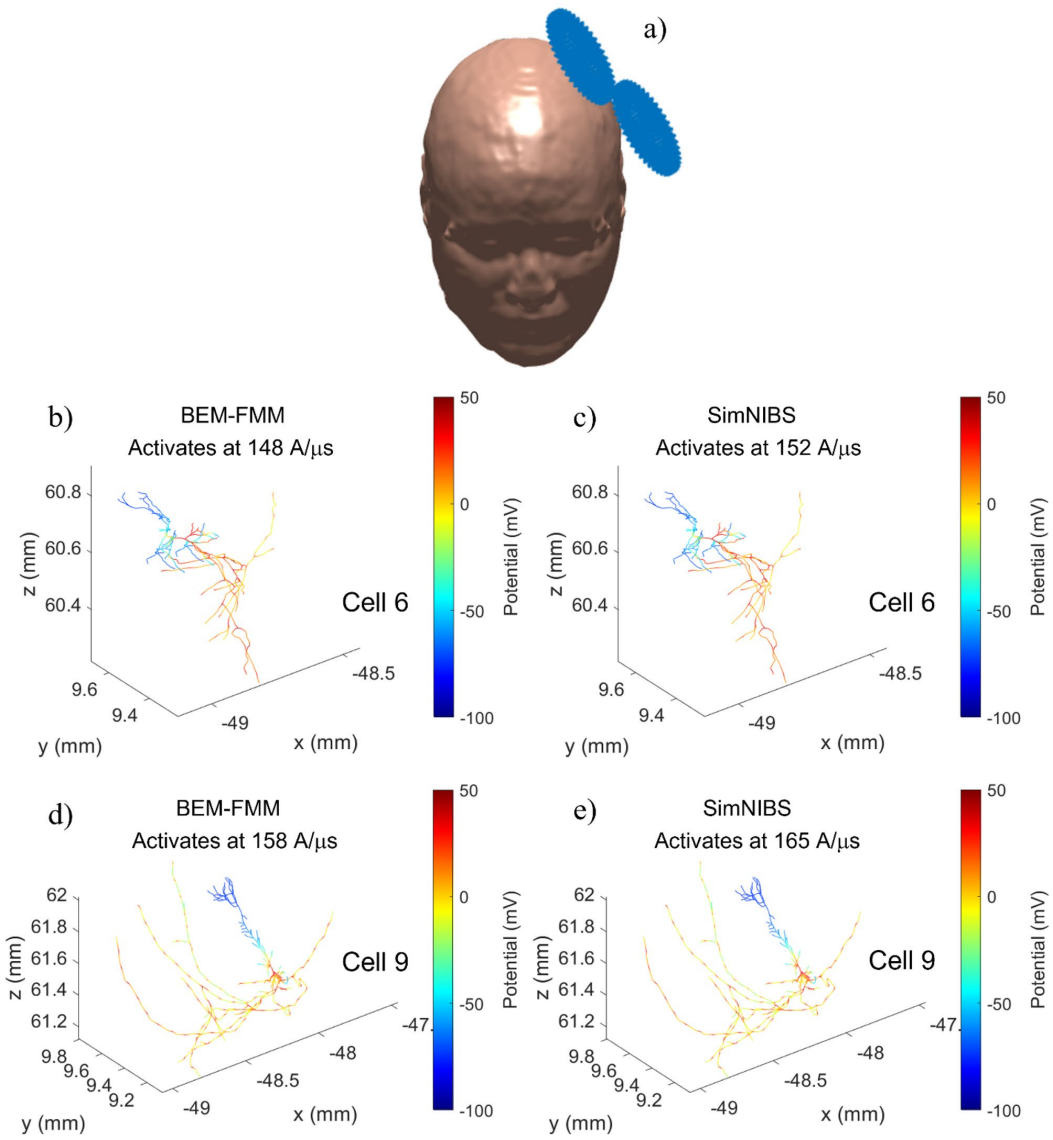
(**a**) Coil position for the Ernie model. (**b**–**e**) activating thresholds and action potentials at 0.7 ms after activation.

Three intracortical neural cells (#4, 6, and 9) from a multi-scale toolbox Neuron Modeling for TMS (NeMo-TMS)^37^ were placed 1 mm below the grey matter surface of the refined Ernie model (0.4 nodes per mm^2^) on the coil axis as shown in Fig. 6a and their activating thresholds were computed as a function of the E-field intensity, $dI/dt$, of a MagStim 70 mm coil (the SimNIBS coil model is used) for the default NeMo-TMS pulse form. Figure 6b–e below compares the activating thresholds computed with BEM-FMM and SimNIBS, respectively, using the corresponding quasi-potentials. It also illustrates the action potentials at the neuron activation for cells 6 and 9, respectively, at 0.7 ms. The activating thresholds differ by 3.2% (cell 4), 2.6% (cell 6), and 4.2% (cell 9) with BEM-FMM always predicting slightly lower threshold values while the action potentials (membrane voltages) at 0.7 ms are hardly distinguishable. For the non-refined model, the agreement is worse (~ 20% on average). The results indicate that ‘inside’ the gray matter the BEM-FMM and SIMNIBS based modeling of the neuronal activation thresholds show robust agreement. On the other hand, axonal activation mechanisms may be influenced by the tissue heterogeneity (that causes charge accumulation on the conductivity boundaries) that may require more detailed analysis of the fields close to the gray-white matter boundaries^38^. Of course, the E-fields on the micro- and mesoscopic level are not fully characterized by the macroscopic model and how to merge the anatomical and histological information across multiple scales is a topic of further research.

### Major limitations of the present direct solution and BEM-FMM in general

As of today, neither BEM-FMM nor FMM LU are in position to handle macroscopic medium anisotropy and are only suitable for modeling the piecewise homogeneous compartments. One way to address this limitation is to use a volumetric integral equation of the standard BEM approach^39, 40^. Other ways are currently under investigation. In any case, a volumetric tetrahedral mesh would indeed be required, at least in the highly anisotropic regions.

If the anisotropic part of the volume conductor model would be perfectly aligned and have equivalent resolution, the error would be on the order of 10% with respect to the isotropic case as shown by our computations for the healthy subject scanned at Max Planck Inst. for Human Cogn. & Brain Sciences Leipzig, Germany. However, estimation of the anisotropy conductivity tensor based on the diffusion tensor imaging data is subject to its own challenges.

The present FMM-LU method has been tested on four multicore (32–56 cores) 2.6 GHz workstations. The minimum required RAM is approximately 64 Gigabytes. The FMM-LU factorization of the head-model specific system matrix requires approximately 40 min. The present method cannot run on a standard laptop.

### Direct extensions of the present FMM-LU solution

When the primary field of a TMS coil is substituted with the primary field of a cortical EEG dipole (or a cluster of these), the current approach can be readily extended to a forward EEG problem, with identical or shorter execution times (5 s or less, as field visualization is not necessary). This presents another potential advantage over the iterative BEM-FMM for EEG^41^, as it results in an approximate tenfold increase in the speed of the forward solution.

## Conclusion

The direct TMS solver suggested in this study can determine global E-fields in modern high-resolution head models in approximately 5 s, which approaches the speed of single-pulse TMS motor mapping. There are no limitations on coil positions, coil types, coil deformations, ROI sizes, and ROI locations. For the standard head model, solver’s numerical accuracy is better than the accuracy of the widely used first-order FEM software when the isotropic head models are used. The FMM LU method can thus be employed to accelerate optimization problems in which a large number of coil configurations have to be simulated and a very large number of field values have to be computed. For example, approximately 24 h would be necessary to accurately compute intracortical fields at 200,000 observation locations anywhere inside the brain volume for 1,000 TMS coil positions and 20 orientations per position.

The method could be readily expanded to other types of brain stimulation. Ref.^42^ presents the standalone downloadable FMM LU code which replicates all data from Fig. 4, along with a short user’s manual. The computational platform is standard MATLAB running on Windows. The code accepts surface head meshes in STL format and is compatible with the coil models from the BEM-FMM package ^10^. Ref.^42^ also includes several movies recorded for examples from Figs. 4 and 5, respectively, and at the identical computational speed.

### Supplementary Information

Supplementary Information.

## Data Availability

The standalone executable computer code that supports the findings of this study along with six short real-time movies is available from repository of Ref.^42^: *TMS FMM-LU March. 2023: Source code in MATLAB and videos*: DropBox: https://www.dropbox.com/sh/ztra43jfj8afh0z/AAAG8mdqyjkQR9UCWYrJQ14Ha?dl=0.

## References

[1] Giuffre A, Kahl CK, Zewdie E, Wrightson JG, Bourgeois A, Condliffe EG, Kirton A (2021). Reliability of robotic transcranial magnetic stimulation motor mapping. J. Neurophysiol..

[2] Kahl CK, Giuffre A, Wrightson JG, Zewdie E, Condliffe EG, MacMaster FP, Kirton A (2022). Reliability of active robotic neuro-navigated transcranial magnetic stimulation motor maps. Exp. Brain Res..

[3] Chen R, Tam A, Bütefisch C, Corwell B, Ziemann U, Rothwell JC, Cohen LG (1998). Intracortical inhibition and facilitation in different representations of the human motor cortex. J. Neurophysiol..

[4] Wagle-Shukla A, Ni Z, Gunraj CA, Bahl N, Chen R (2009). Effects of short interval intracortical inhibition and intracortical facilitation on short interval intracortical facilitation in human primary motor cortex. J. Physiol..

[5] Daneshzand M, Makarov SN, de Lara LIN, Guerin B, McNab J, Rosen BR, Hämäläinen MS, Raij T, Nummenmaa A (2021). Rapid computation of TMS-induced E-fields using a dipole-based magnetic stimulation profile approach. NeuroImage.

[6] Gomez LJ, Dannhauer M, Peterchev AV (2021). Fast computational optimization of TMS coil placement for individualized electric field targeting. NeuroImage..

[7] Wang D, Hasan NI, Dannhauer M, Yucel AC, Gomez L (2023). Fast computational E-field dosimetry for transcranial magnetic stimulation using adaptive cross approximation and auxiliary dipole method (ACA-ADM). NeuroImage.

[8] Weise K, Wartman WA, Knösche TR, Nummenmaa AR, Makarov SN (2022). The effect of meninges on the electric fields in TES and TMS. Numerical modeling with adaptive mesh refinement. Brain Stimul..

[9] Makarov SN, Wartman WA, Noetscher GM, Fujimoto K, Zaidi T, Burnham EH, Daneshzand M, Nummenmaa A (2021). Degree of improving TMS focality through a geometrically stable solution of an inverse TMS problem. NeuroImage.

[10] Makarov SN, Wartman WA, Daneshzand M, Fujimoto K, Raij T, Nummenmaa A (2020). A software toolkit for TMS electric-field modeling with boundary element fast multipole method: An efficient MATLAB implementation. J. Neural Eng..

[11] Makarov SN, Noetscher GM, Raij T, Nummenmaa A (2018). A quasi-static boundary element approach with fast multipole acceleration for high-resolution bioelectromagnetic models. IEEE Trans. Biomed. Eng..

[12] Sushnikova, D., Greengard, L., O’Neil, M., & Rachh, M. FMM-LU: A fast direct solver for multiscale boundary integral equations in three dimensions. arXiv pre-print https://arxiv.org/pdf/2201.07325.pdf (2022).

[13] Weise K, Numssen O, Kalloch B, Zier AL, Thielscher A, Haueisen J, Hartwigsen G, Knösche TR (2023). Precise motor mapping with transcranial magnetic stimulation. Nat. Protoc..

[14] Aonuma S, Gomez-Tames J, Laakso I, Hirata A, Takakura T, Tamura M, Muragaki Y (2018). A high-resolution computational localization method for transcranial magnetic stimulation mapping. NeuroImage.

[15] Lynch CJ, Elbau IG, Ng TH, Wolk D, Zhu S, Ayaz A, Power JD, Zebley B, Gunning FM, Liston C (2022). Automated optimization of TMS coil placement for personalized functional network engagement. Neuron.

[16] Beatson R, Greengard L, Ainsworth M (1997). A short course on fast multipole methods. Wavelets, Multilevel Methods, and Elliptic PDEs.

[17] Greengard L, Rokhlin V (1997). A new version of the Fast Multipole Method for the Laplace equation in three dimensions. Acta Numer..

[18] Fischl B (2012). FreeSurfer. NeuroImage.

[19] FreeSurfer Software Suite 2022. https://www.zotero.org/freesurfer.

[20] *Structural Brain Mapping Group. Computational Anatomy Toolbox (CAT)*. Univ. of Jena, Germany. Accessed 04/05/21. http://www.neuro.uni-jena.de/wordpress/publications/

[21] Greengard L, Gueyffier D, Martinsson P-G, Rokhlin V (2009). Fast direct solvers for integral equations in complex three-dimensional domains. Acta Numer..

[22] Minden V, Damle A, Ho KL, Ying L (2016). A technique for updating hierarchical skeletonization-based factorizations of integral operators. Multiscale Model. Simul..

[23] Vladimirov VS (1971). Equations of Mathematical Physics.

[24] Kress, R. *Linear Integral Equations* 3rd edn, Vol. 82 (Springer, New York, 2014).

[25] Htet AT, Saturnino GB, Burnham EH, Noetscher GM, Nummenmaa A, Makarov SN (2019). Comparative performance of the finite element method and the boundary element fast multipole method for problems mimicking transcranial magnetic stimulation (TMS). J. Neural Eng..

[26] Wartrnan, W. A., Burnham, E. H., Makarov, S. N., Davids, M., Daneshzand, M., Nummenmaa, A. High resolution computational modeling of transcranial stimulation using the MIDA Head Model. In *2021 10th International IEEE/EMBS Conference on Neural Engineering (NER), Italy*, pp. 1044–1047 (2021). 10.1109/NER49283.2021.9441170

[27] Gimbutas, Z., Greengard, L., Magland, J., Rachh, M., & Rokhlin, V. *fmm3D Documentation.* Release 0.1.0. 2019–2022. https://github.com/flatironinstitute/FMM3D & https://github.com/flatironinstitute/FMM3D/blob/master/fmm3d_manual.pdf.

[28] Saad Y (2003). Iterative Methods for Sparse Linear Systems.

[29] Greengard L, Rokhlin V (1987). A fast algorithm for particle simulations. J. Comput. Phys..

[30] Thielscher A, Antunes A, Saturnino GB (2015). Field modeling for transcranial magnetic stimulation: A useful tool to understand the physiological effects of TMS?. Annu. Int. Conf. IEEE Eng. Med. Biol. Soc..

[31] Saturnino, G.B., Puonti, O., Nielsen, J. D., Antonenko, D., Madsen, K. H., Thielscher, A. SimNIBS 2.1: A Comprehensive Pipeline for Individualized Electric Field Modelling for Transcranial Brain Stimulation. 2019. In: Makarov S, Horner M, Noetscher G, editors. *Brain and Human Body Modeling: Computational Human Modeling at EMBC 2018*. Springer; 2019. Chapter 1. PMID: 31725247. 31725247

[32] Penny W, Friston K, Ashburner J, Kiebel S, Nichol T (2007). Statistical Parametric Mapping: The Analysis of Functional Brain Images.

[33] Van Essen DC, Ugurbil K, Auerbach E, Barch D, Behrens TE, Bucholz R, Chang A, Chen L, Corbetta M, Curtiss SW, Della Penna S, Feinberg D, Glasser MF, Harel N, Heath AC, Larson-Prior L, Marcus D, Michalareas G, Moeller S, Oostenveld R, Petersen SE, Prior F, Schlaggar BL, Smith SM, Snyder AZ, Xu J, Yacoub E (2012). The human connectome project: A data acquisition perspective. NeuroImage.

[34] Raffin E, Pellegrino G, Di Lazzaro V, Thielscher A, Siebner HR (2015). Bringing transcranial mapping into shape: Sulcus-aligned mapping captures motor somatotopy in human primary motor hand area. NeuroImage.

[35] Makarov SN, Navarro de Lara L, Noetscher GM, Nummenmaa A (2019). Modeling Primary Fields of TMS Coils with the Fast Multipole Method. bioRxiv.

[36] Gomez LJ, Dannhauer M, Koponen LM, Peterchev AV (2019). Conditions for numerically accurate TMS electric field simulation. Brain Stimul..

[37] Shirinpour S, Hananeia N, Rosado J, Tran H, Galanis C, Vlachos A, Jedlicka P, Queisser G, Opitz A (2021). Multi-scale modeling toolbox for single neuron and subcellular activity under Transcranial Magnetic Stimulation. Brain Stimul..

[38] Miranda PC, Correia L, Salvador R, Basser PJ (2007). Tissue heterogeneity as a mechanism for localized neural stimulation by applied electric fields. Phys. Med. Biol..

[39] Gomez LJ, Yücel AC, Michielssen E (2018). The ICVSIE: A general purpose integral equation method for bio-electromagnetic analysis. IEEE Trans. Biomed. Eng..

[40] Rahmouni L, Mitharwal R, Andriulli FP (2017). Two volume integral equations for the inhomogeneous and anisotropic forward problem in electroencephalography. J. Comput. Phys..

[41] Makarov SN, Hämäläinen M, Okada Y, Noetscher GM, Ahveninen J, Nummenmaa A (2020). Boundary element fast multipole method for enhanced modeling of neurophysiological recordings. IEEE Trans. Biomed. Eng..

[42] *TMS FMM-LU Jan-Feb. 2023: Source code in MATLAB and videos*. DropBox: https://www.dropbox.com/sh/ztra43jfj8afh0z/AAAG8mdqyjkQR9UCWYrJQ14Ha?dl=0

