# Supplementary material for: A fast direct solver for surface-based whole-head modeling of transcranial magnetic stimulation
Source: Sci Rep. 2023 Oct 31;13:18657. doi: 10.1038/s41598-023-45602-5 (PMC10618282; doi:10.1038/s41598-023-45602-5)
Supplement: Supplementary file 1 — Supplementary Information. [file 41598_2023_45602_MOESM1_ESM.docx]

Our paper is***not***reporting experiments on humans and/or the use of human tissue samples. We run simulations on a publicly available Connectome dataset [1].

1. Van Essen DC, Ugurbil K, Auerbach E, Barch D, Behrens TE, Bucholz R, Chang A, Chen L, Corbetta M, Curtiss SW, Della Penna S, Feinberg D, Glasser MF, Harel N, Heath AC, Larson-Prior L, Marcus D, Michalareas G, Moeller S, Oostenveld R, Petersen SE, Prior F, Schlaggar BL, Smith SM, Snyder AZ, Xu J, Yacoub E. The Human Connectome Project: A data acquisition perspective*. NeuroImage*. 2012; 62(4):2222–2231. Online (Jan. 2020): <http://www.humanconnectomeproject.org/>
